# Bilateral Symmetric Heterotopic Ossification of the Pubic Bones in a Former Footballer: A Case Report

**DOI:** 10.7759/cureus.116861

**Published:** 2026-09-24

**Authors:** Xavier Penda, Taku Angwa Neba, Henry Ndasi

**Affiliations:** 1 Orthopaedics and Traumatology, Mutengene Christian Orthopedic Residency (MCORE) Program, Mutengene, CMR; 2 Orthopaedics and Trauma, Baptist Hospital Mutengene, Mutengene, CMR

**Keywords:** adductor muscles, football, heterotopic ossification, pubic bone, sports injury

## Abstract

Heterotopic ossification (HO) is the formation of extraskeletal bone within soft tissue, most often arising after trauma, surgery, burns or neurological injury. It is typically described around the hip, elbow, shoulder and knee, and reports of HO centred on the pubic bones are exceptional. We report a 39-year-old former footballer who presented with a two-year history of insidious, activity-related pelvic and groin pain, without any recollected acute injury. Examination revealed bilateral, symmetric, bony-hard groin masses that were tender on palpation, with a full and symmetric range of hip motion, including abduction, and an intact distal neurovascular status. Pelvic radiography demonstrated bilateral, well-defined, horn-like ossific projections arising from the medial aspects of the superior pubic rami, and three-dimensional computed tomography confirmed symmetric exuberant ossification within the adductor musculature along the anterior aspect of both pubic bones. Osteochondroma, osteoma and chondroma were considered preoperatively. Both lesions were excised through an anterior approach; each was continuous with the pubic cortex through a broad bony base. Histopathology showed fragments of benign bone with fatty marrow and scant hyaline cartilage, with no evidence of malignancy; these features were reported as non-specific and requiring radiologic correlation. Integrating the imaging and histological findings, a diagnosis of bilateral HO of the pubic bones was made. At 16 months the patient was pain-free with no palpable mass and had returned to recreational football. This case illustrates that HO may arise at the adductor origin of the pubic bones in the absence of a discrete traumatic event, that it can closely mimic osteochondroma both radiologically and histologically, and that radiologic-pathologic correlation is essential to avoid misdiagnosis.

## Introduction

Heterotopic ossification (HO) is a complex pathological process marked by the formation of extraskeletal bone in soft tissue [1,2]. Two broad forms are recognised: an acquired form that follows trauma, surgery, burns or neurological injury (traumatic HO, tHO), which is by far the more common, and a rare, genetically driven form, fibrodysplasia ossificans progressiva (FOP) [3,4]. The burden of the acquired form is considerable in high-energy injury; among combat-wounded patients requiring orthopaedic intervention, an HO rate of 64.5% has been reported [5]. By contrast, FOP is exceedingly rare, with an estimated prevalence of approximately 0.88 cases per million people [6].

The pathogenesis differs between the two forms. FOP is caused by a recurrent gain-of-function mutation in the ACVR1 gene, which constitutively activates bone morphogenetic protein signalling and drives progressive ectopic endochondral bone formation [6,7]. Acquired HO, in contrast, is best understood as a dysregulated tissue-repair response, in which local injury, inflammation and altered mechanical loading recruit mesenchymal progenitors that undergo inappropriate osteogenic differentiation within soft tissue [1-3]. Recognised risk factors for acquired HO include a prior or family history of HO, trauma and surgery, hypertrophic osteoarthritis, ankylosing spondylitis, traumatic brain and spinal cord injury, prolonged mechanical ventilation, and male sex [3,4,8].

Clinically, HO may present with soft-tissue or joint pain, a palpable mass, joint stiffness and restricted range of motion [1,3,4,9]. Plain radiography, computed tomography (CT) and magnetic resonance imaging assist both diagnosis and perioperative planning [3,9]. Management is initially conservative and aims to limit the early inflammatory phase, using non-steroidal anti-inflammatory drugs (NSAIDs) such as indomethacin or celecoxib, bisphosphonates, and radiotherapy; excision is generally deferred until the ectopic bone has matured, typically six to 12 months after onset [3,4,9]. Reported anatomical sites are dominated by the hip, elbow, shoulder and knee [1,2,9], and although HO has been described within the adductor compartment after acute injury [10,11], involvement centred on the pubic bones is distinctly uncommon.

While HO is classically described as bone formation within “soft tissue”, we present a rare and atypical case in which mature ectopic bone arose bilaterally and symmetrically from the pubic bones at the adductor origin, in a former footballer with no recollected acute injury. This case report was prepared in accordance with the CARE guidelines [12]. 

## Case presentation

A 39-year-old man, a former footballer, presented with pelvic pain of approximately two years' duration. The pain began insidiously, was sharp and non-radiating. It was intermittent, was aggravated by strenuous activity such as playing a football match and was relieved by over-the-counter analgesics. He denied any direct trauma preceding the onset of pain. The symptoms had made playing football progressively less enjoyable, which prompted consultation at our orthopaedic surgery department, where he was assessed and managed throughout. There was no unintended weight loss, night sweats, persistent fever or unrelenting fatigue. He reported regular alcohol consumption, did not use tobacco, and was on no routine medications. He reported no family history of a similar presentation and no personal or family history of previous ectopic bone formation.

On examination he was conscious, oriented and appeared well, with vital signs within normal limits; the conjunctivae were pink and the sclerae anicteric. Bilateral symmetric swellings were present in both groins, with bony-hard palpable masses that were tender on palpation and not fixed to the overlying skin. The range of motion of both hip joints, including abduction and adduction, was full, symmetric and painless, and the distal neurovascular status of both lower limbs was intact. There was no local warmth, erythema or overlying skin change. The remainder of the examination was unremarkable.

An anteroposterior radiograph of the pelvis demonstrated bilateral, well-defined, horn-like ossific lesions projecting from the medial aspects of the superior pubic rami (Figure 1). Three-dimensional CT reconstruction confirmed symmetric, exuberant ossification within the adductor musculature along the anterior aspect of both pubic bones (Figure 2). On independent review, the reporting radiologist described the appearances as symmetric exuberant HO of the adductor musculature along the anterior aspect of both pubic bones, considered definitely benign, with no evidence of osteochondroma, and favoured sequelae of old injury or trauma.

**Figure 1:**
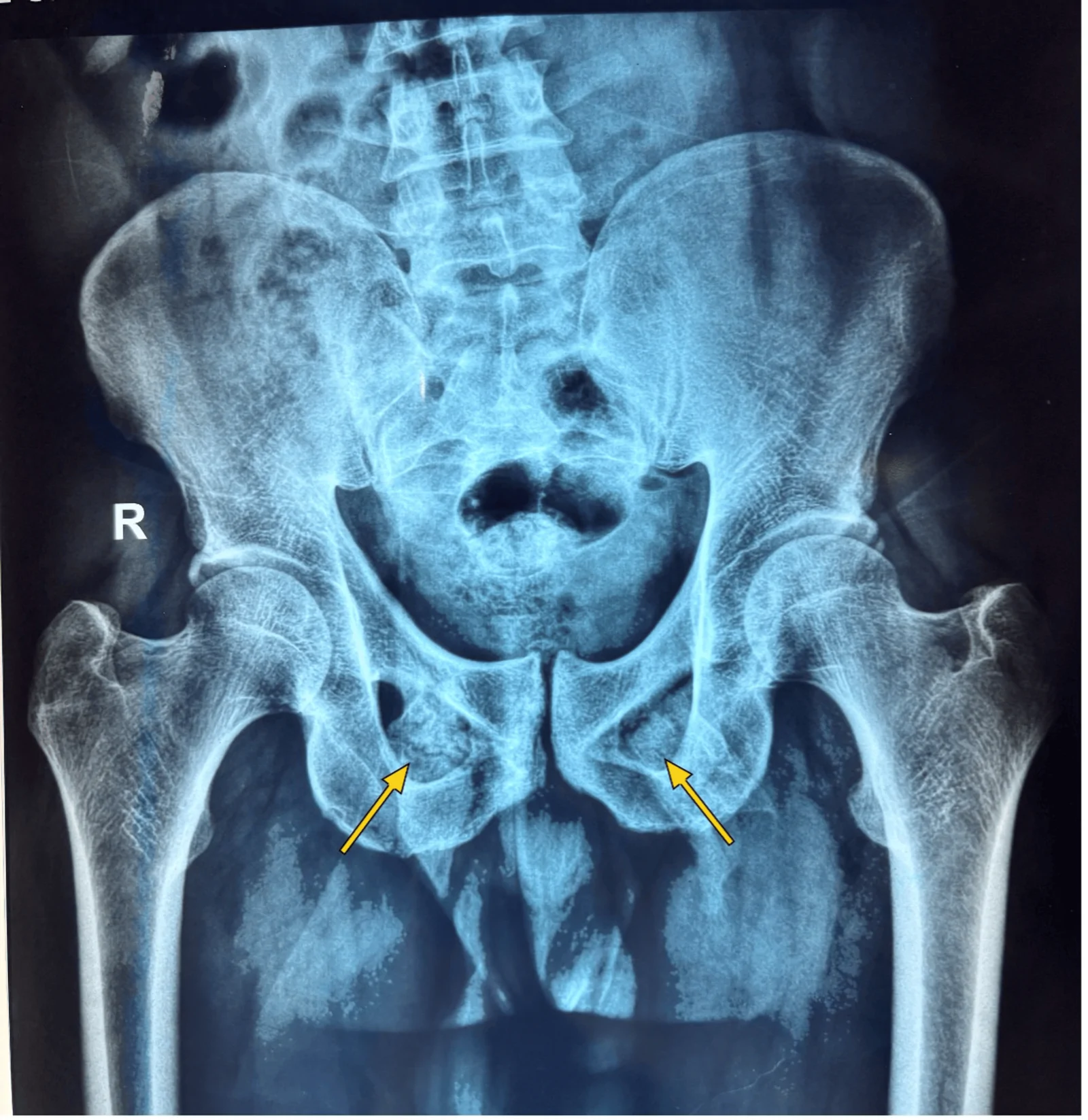
Anteroposterior radiograph of the pelvis showing bilateral, well-defined, horn-like ossific lesions arising from the medial aspects of the superior pubic rami. Arrows indicate the ossific lesions on each side

**Figure 2:**
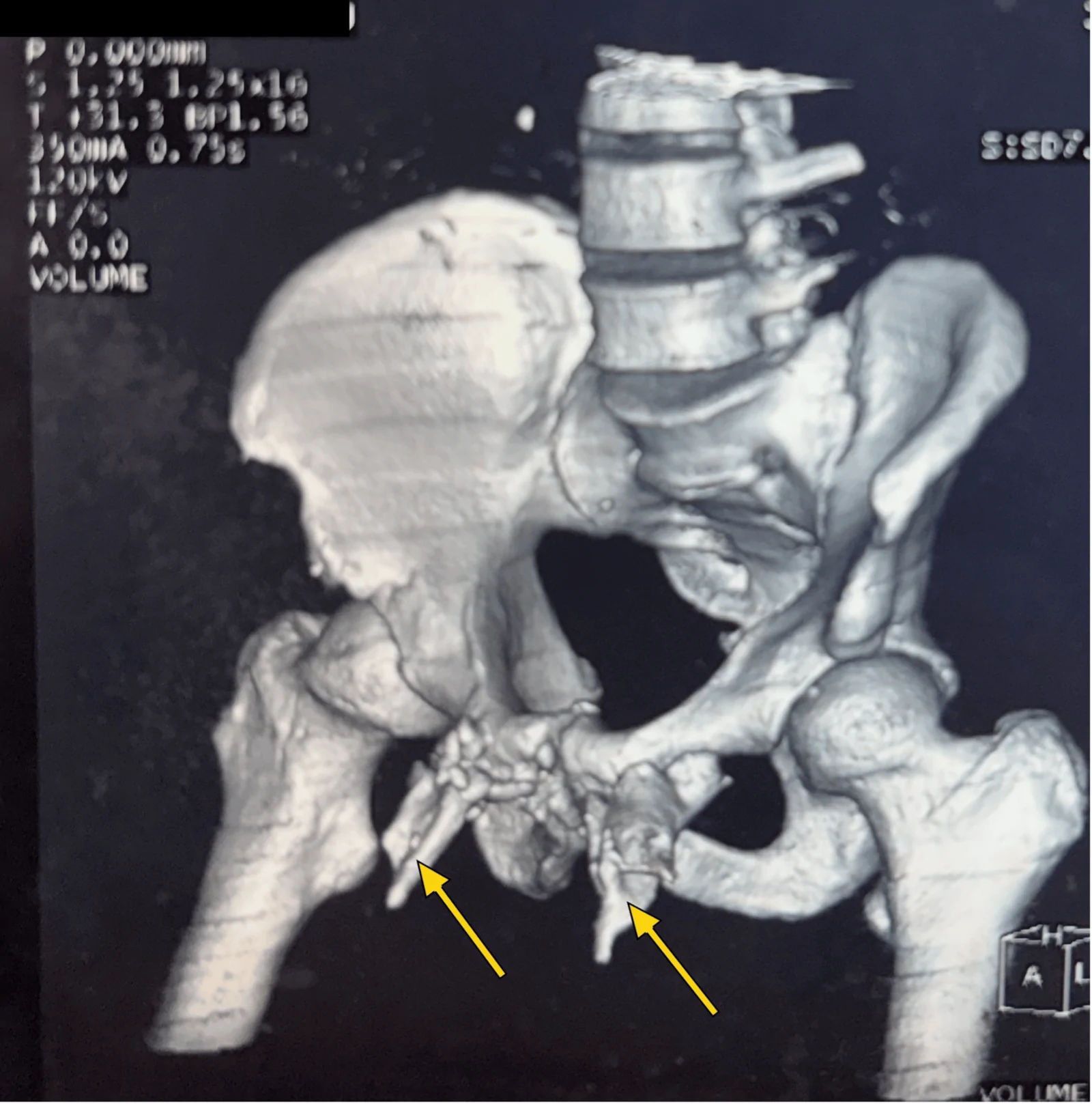
Three-dimensional computed tomography reconstruction of the pelvis demonstrating symmetric exuberant heterotopic ossification within the adductor musculature along the anterior aspect of both pubic bones. Arrows indicate the ossific lesions on each side

The preoperative working diagnosis was HO, with osteochondroma, osteoma and chondroma as the main differential diagnoses. Osteochondroma was considered because each lesion projected from the surface of the pubis, but the lesions were bilateral and symmetric, and on CT neither showed continuity with the pubic medullary cavity, the defining feature of an osteochondroma. Osteoma was unlikely because osteomas consist of dense ivory bone and arise mostly in the craniofacial skeleton, whereas these lesions were mature trabecular bone at a muscle origin. Chondroma was unlikely in the absence of chondroid matrix mineralisation or cortical scalloping on imaging. Because of persistent activity-limiting pain and diagnostic uncertainty, excisional biopsy was undertaken after the patient gave informed consent. The surgery was done three months following the initial consultation. Through an anterior approach to the pelvis, both lesions were exposed and excised. Intraoperatively, each mass was continuous with the cortex of the pubic bone through a broad bony base, without a discernible cleavage plane, requiring osteotomy at the base to achieve excision; the surrounding adductor musculature was otherwise unremarkable (Figure 3). The adductor origins overlying each lesion were mostly elevated from the mass with a periosteal elevator, although a small portion of the insertion was partially detached during exposure; the remainder of the insertion was left in continuity with the pubic cortex. The osteotomy was deliberately performed outside the tendinous footprint, so that no tendon required formal detachment or reattachment. The excised fragments measured up to approximately 7.5 × 2.5 cm.

**Figure 3:**
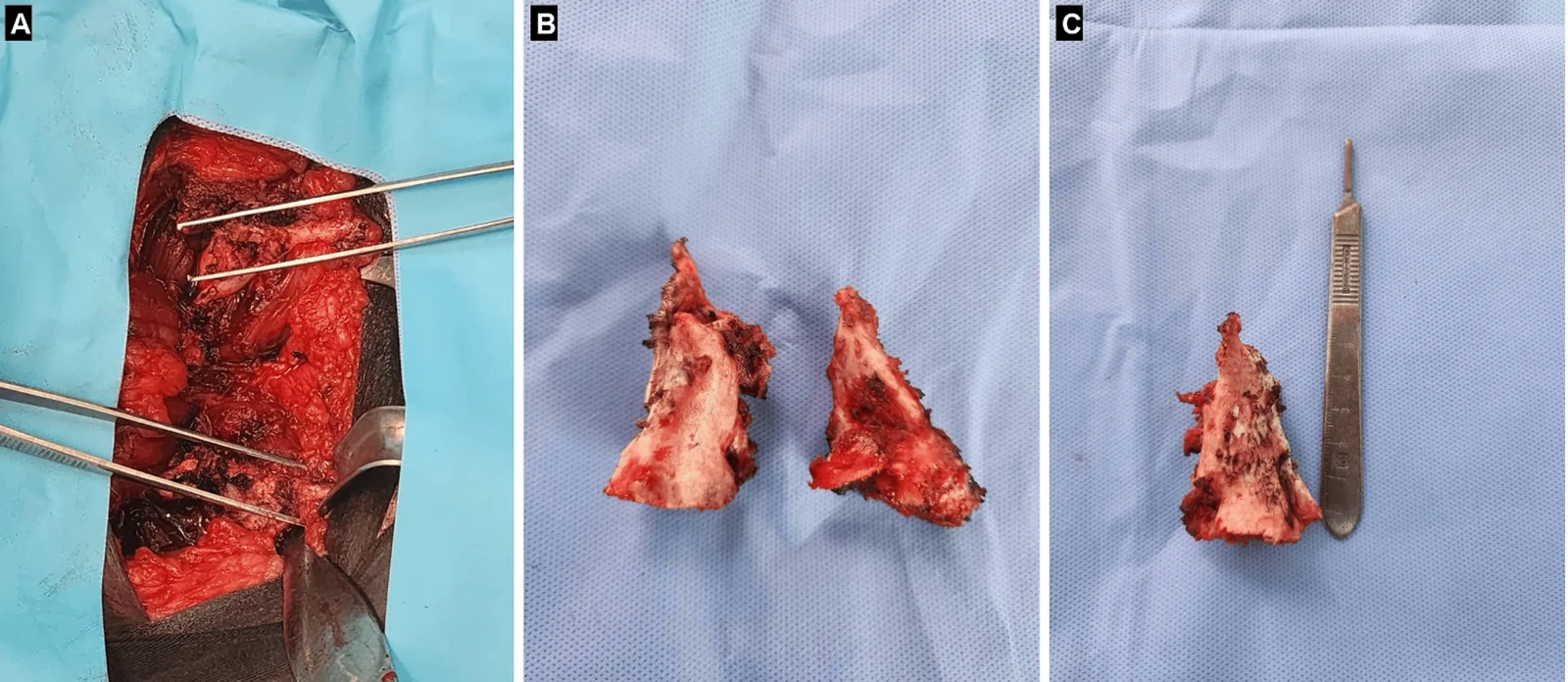
Intraoperative and specimen photographs. (A) Anterior approach to the pelvis with the ossified mass exposed at the adductor origin. (B) Both excised lesions. (C) Excised lesion alongside a scalpel handle for scale.

Histopathological examination showed fragments of benign bone with fatty marrow; rare fragments contained portions of hyaline cartilage, and there was no evidence of malignancy (Figure 4). These histological elements are not specific, and identical fragments may be seen in osteochondroma. The diagnosis therefore rested on radiologic-pathologic correlation. Three findings excluded osteochondroma. The ossification was bilateral and symmetric and lay along the adductor musculature rather than forming a solitary stalked exostosis. The lesions showed no continuity with the pubic medullary cavity. The cartilage in the specimen was scant and fragmentary, with no cartilage cap showing organised endochondral maturation. Taken with the absence of malignancy, these findings established the diagnosis of mature HO at the adductor origin of the pubic bones.

**Figure 4:**
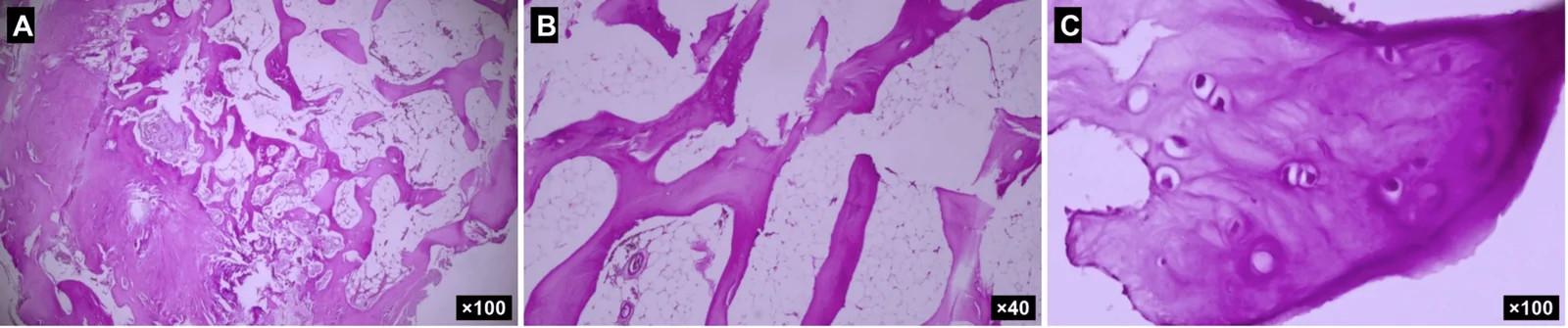
Histopathological appearances (haematoxylin and eosin). (A, B) Benign bone, trabeculae of lamellar bone with hypocellular marrow spaces (A, original magnification ×100; B, ×40). (C) A fragment of hyaline cartilage (×100). No cytological atypia.

The postoperative course was uneventful and the patient was discharged home on a one-week course of an NSAID (diclofenac) for analgesia. He was allowed to bear weight as tolerated from the first postoperative day, with no restriction of hip movement. He was seen at six weeks with only minor pain and needed no rehabilitation. At follow-up one year and four months after surgery he was well, with no pain and no palpable mass, and he had resumed sporting activity, although not at a professional level. Follow-up was clinical; no surveillance radiographs were obtained.

From the patient's perspective, he was very satisfied with the outcome of surgery. He reported that the resolution of his pain and, above all, his ability to resume his work were the most important gains for him, and he was pleased to have returned to playing recreational football.

## Discussion

HO is a well-characterised complication of trauma, surgery, burns and central neurological injury, and is most frequently encountered around the hip, elbow, shoulder and knee [1,2,9]. The present case is unusual in three respects: the lesions were centred on the pubic bones at the adductor origin, they were bilateral and strikingly symmetric, and they arose without any recollected acute injury. To our knowledge, bilateral symmetric HO of the pubic bones presenting as painful groin masses in an athlete has rarely been described.

The most plausible mechanism in this patient is repetitive microtrauma at the adductor enthesis. The adductor longus, adductor brevis and gracilis originate from the anterior aspect of the pubic body and inferior pubic ramus, precisely where the ossification was located, and these origins are subjected to substantial and repeated loading during kicking, sprinting and pivoting. Football is therefore associated with a high burden of adductor and groin pathology. Ossification of the adductor compartment is recognised after acute injury: Rajakulendran and Field described late heterotopic bone formation following an adductor origin avulsion injury [10], and de Smet et al. reported post-traumatic myositis ossificans of the adductor longus in a footballer that required surgical excision after failed conservative treatment [11]. Our patient denied a discrete injury, but repeated subclinical enthesopathic microtrauma accumulated over a playing career offers a coherent explanation, and would also account for the symmetry, since both limbs are loaded comparably in football. Notably, the reporting radiologist independently favoured sequelae of old injury or trauma. It is also relevant that non-traumatic myositis ossificans circumscripta is well recognised as a diagnostic trap that can mimic neoplasia when no injury is recalled [13].

The differential diagnosis of a mineralised mass arising from the pubic bone in an adult merits emphasis, because both benign neoplasia and malignancy must be excluded. Osteochondroma is the principal differential diagnosis, and was the main alternative considered here; it is a cartilage-capped bony projection that characteristically shows medullary continuity with the parent bone, and although the pelvis is an uncommon site, pelvic and pubic examples are described [14]. Distinguishing osteochondroma from HO matters clinically, since pelvic exostoses carry a recognised, if small, risk of secondary chondrosarcomatous transformation, and lesions of the pelvis and pubic region warrant particular caution [15]. Other considerations include osteoma, parosteal or juxtacortical lesions, traction enthesophytes at the adductor origin, sequelae of osteitis pubis or athletic pubalgia, and, importantly, parosteal osteosarcoma, which can mimic benign juxtacortical ossification. Reassuring features in our patient were the bilateral symmetry, sharply defined margins, mature and organised ossification on CT, absence of cortical destruction or a soft-tissue mass, and the absence of constitutional symptoms.

This case also illustrates a genuine diagnostic pitfall in radiologic-pathologic correlation. The excised tissue showed benign lamellar bone with fatty marrow and scant hyaline cartilage. These appearances are non-specific and could equally be seen in osteochondroma. Three findings nevertheless excluded that diagnosis. First, the imaging showed bilateral, symmetric ossification distributed along the adductor musculature rather than a solitary, stalked, cartilage-capped exostosis. Second, each lesion lay on the surface of the pubic cortex without continuity with the medullary cavity, whereas medullary continuity with the parent bone is the defining feature of an osteochondroma [16]. Third, in osteochondroma the cartilage cap is a continuous, organised surface layer showing endochondral maturation, whereas the cartilage in our specimen was scant and fragmentary. Taken together, these findings are diagnostic of HO, and a non-specific histological report does not weaken that conclusion.

Management of established HO is guided by symptoms and lesion maturity. Conservative measures, including NSAIDs, bisphosphonates and radiotherapy, target the early inflammatory phase, whereas surgery is reserved for mature, symptomatic lesions causing pain, mass effect or functional limitation, generally after six to 12 months [3,4,9]. Our patient underwent excision because of activity-limiting pain of two years' duration and unresolved diagnostic uncertainty, and surgery served both therapeutic and diagnostic purposes. Recurrence is the principal long-term concern after excision; combined excision and postoperative NSAID prophylaxis has been associated with low recurrence rates and good functional results in hip HO [17], and radiotherapy or extended NSAID courses are used in selected high-risk cases. Our patient received only a short analgesic course of diclofenac rather than a formal prophylactic regimen, yet remained clinically free of pain and of any palpable mass at 16 months. This may reflect a lower recurrence risk for mature, mechanically driven ossification at an enthesis in a patient without neurological injury, although it cannot be generalised from a single case.

A further question is whether a predisposition to HO confers any increased risk of subsequently developing a musculoskeletal neoplasm. No such association has been established. HO, including myositis ossificans, is regarded as a benign, self-limiting metaplastic process, and neither large series of acquired HO after trauma and arthroplasty nor natural history studies of FOP report an excess of bone or soft-tissue tumours [1-3,6].

The limitations of this report should be acknowledged. No biochemical investigations, such as alkaline phosphatase, calcium, phosphate or inflammatory markers, were performed; these would not have been diagnostic but would have strengthened the exclusion of metabolic bone disease. Magnetic resonance imaging was not obtained and might have better characterised the adductor enthesis and any cartilage cap. Finally, follow-up was clinical only. Surveillance radiographs were not obtained because the patient remained completely symptom-free, with no pain and no palpable mass. Recurrent ectopic bone is usually detectable radiographically before it becomes clinically apparent, so subclinical radiographic recurrence cannot be excluded in this patient. The follow-up period of 16 months, while informative, also remains relatively short.

## Conclusions

HO should be considered in the differential diagnosis of a bony-hard groin mass arising from the pubic bone, including in patients who recall no acute injury. In athletes, repetitive microtrauma at the adductor enthesis provides a plausible mechanism and may account for bilateral, symmetric disease. This case highlights that mature HO can closely mimic osteochondroma both radiologically and histologically, and that a non-specific histological report does not exclude the diagnosis; correlation of imaging, operative and pathological findings is essential. In a symptomatic patient with mature ectopic bone, excision achieved durable relief of pain and a return to recreational sport, with no clinical recurrence at 16 months.

## References

[1] Xu Y, Huang M, He W (2022). Heterotopic ossification: clinical features, basic researches, and mechanical stimulations. Front Cell Dev Biol.

[2] Meyers C, Lisiecki J, Miller S (2019). Heterotopic ossification: a comprehensive review. JBMR Plus.

[3] Hwang CD, Pagani CA, Nunez JH (2022). Contemporary perspectives on heterotopic ossification. JCI Insight.

[4] Lawand J, Loeffelholz Z, Khurshid B, Barcak E (2023). Heterotopic ossification after trauma. Orthop Clin North Am.

[5] Forsberg JA, Pepek JM, Wagner S (2009). Heterotopic ossification in high-energy wartime extremity injuries: prevalence and risk factors. J Bone Joint Surg Am.

[6] Pignolo RJ, Hsiao EC, Baujat G, Lapidus D, Sherman A, Kaplan FS (2021). Prevalence of fibrodysplasia ossificans progressiva (FOP) in the United States: estimate from three treatment centers and a patient organization. Orphanet J Rare Dis.

[7] Guan Y, Ma D (2024). Fibrodysplasia ossificans progressiva complicated with post traumatic and infectious myositis ossificans in masseter: a case report. Medicine (Baltimore).

[8] Firoozabadi R, O'Mara TJ, Swenson A, Agel J, Beck JD, Routt M (2014). Risk factors for the development of heterotopic ossification after acetabular fracture fixation. Clin Orthop Relat Res.

[9] Vanden Bossche L, Vanderstraeten G (2005). Heterotopic ossification: a review. J Rehabil Med.

[10] Rajakulendran K, Field RE (2012). Late formation of heterotopic bone following an adductor origin avulsion injury. Injury.

[11] de Smet GH, Buijk SE, Weir A (2020). Surgical excision of post-traumatic myositis ossificans of the adductor longus in a football player. BMJ Case Rep.

[12] Gagnier JJ, Kienle G, Altman DG, Moher D, Sox H, Riley D (2013). The CARE guidelines: consensus-based clinical case reporting guideline development. Glob Adv Health Med.

[13] El Bardouni A, Boufettal M, Zouaidia F, Kharmaz M, Berrada MS, Mahassini N, El Yaacoubi M (2014). Non-traumatic myositis ossificans circumscripta: a diagnosis trap. J Clin Orthop Trauma.

[14] Ratra R, Peshin C (2020). Rare presentation of benign osteochondroma: a case report. J Orthop Case Rep.

[15] Nystrom LM, DeYoung BR, Morcuende JA (2013). Secondary chondrosarcoma of the pelvis arising from a solitary exostosis in an 11-year-old patient: a case report with 5-year follow-up. Iowa Orthop J.

[16] Murphey MD, Choi JJ, Kransdorf MJ, Flemming DJ, Gannon FH (2000). Imaging of osteochondroma: variants and complications with radiologic-pathologic correlation. Radiographics.

[17] Wick M, Müller EJ, Hahn MP, Muhr G (1999). Surgical excision of heterotopic bone after hip surgery followed by oral indomethacin application: is there a clinical benefit for the patient?. Arch Orthop Trauma Surg.

